# Differential Impact of Severity and Duration of *Status Epilepticus*, Medical Countermeasures, and a Disease-Modifier, Saracatinib, on Brain Regions in the Rat Diisopropylfluorophosphate Model

**DOI:** 10.3389/fncel.2021.772868

**Published:** 2021-10-15

**Authors:** Meghan Gage, Marson Putra, Crystal Gomez-Estrada, Madison Golden, Logan Wachter, Megan Gard, Thimmasettappa Thippeswamy

**Affiliations:** ^1^Department of Biomedical Sciences, College of Veterinary Medicine, Iowa State University, Ames, IA, United States; ^2^Neuroscience Interdepartmental Program, Iowa State University, Ames, IA, United States

**Keywords:** organophosphate toxicity, status epilepticus severity, gliosis, neurodegeneration, disease mitigation, saracatinib (AZD0530)

## Abstract

Acute organophosphate (OP) toxicity poses a significant threat to both military and civilian personnel as it can lead to a variety of cholinergic symptoms including the development of *status epilepticus* (SE). Depending on its severity, SE can lead to a spectrum of neurological changes including neuroinflammation and neurodegeneration. In this study, we determined the impact of SE severity and duration on disease promoting parameters such as gliosis and neurodegeneration and the efficacy of a disease modifier, saracatinib (AZD0530), a Src/Fyn tyrosine kinase inhibitor. Animals were exposed to 4 mg/kg diisopropylfluorophosphate (DFP, s.c.) followed by medical countermeasures. We had five experimental groups: controls (no DFP), animals with no continuous convulsive seizures (CS), animals with ∼20-min continuous CS, 31-60-min continuous CS, and > 60-min continuous CS. These groups were then assessed for astrogliosis, microgliosis, and neurodegeneration 8 days after DFP exposure. The 31-60-min and > 60-min groups, but not ∼20-min group, had significantly upregulated gliosis and neurodegeneration in the hippocampus compared to controls. In the piriform cortex and amygdala, however, all three continuous CS groups had significant upregulation in both gliosis and neurodegeneration. In a separate cohort of animals that had ∼20 and > 60-min of continuous CS, we administered saracatinib for 7 days beginning three hours after DFP. There was bodyweight loss and mortality irrespective of the initial SE severity and duration. However, in survived animals, saracatinib prevented spontaneous recurrent seizures (SRS) during the first week in both severity groups. In the ∼20-min CS group, compared to the vehicle, saracatinib significantly reduced neurodegeneration in the piriform cortex and amygdala. There were no significant differences in the measured parameters between the naïve control and saracatinib on its own (without DFP) groups. Overall, this study demonstrates the differential effects of the initial SE severity and duration on the localization of gliosis and neurodegeneration. We have also demonstrated the disease-modifying potential of saracatinib. However, its’ dosing regimen should be optimized based on initial severity and duration of CS during SE to maximize therapeutic effects and minimize toxicity in the DFP model as well as in other OP models such as soman.

## Introduction

Organophosphate nerve agents (OPNAs) have historically been used in chemical warfare scenarios to induce a variety of cholinergic symptoms (Coupland and Leins, 2005; Jett, 2007; Watson et al., 2015; Mukherjee and Gupta, 2020). OPNAs include the G-series, V-series, GV-series, and Novichek series (Mukherjee and Gupta, 2020). In 2013, Sarin, a G-series agent, was used as a chemical weapon and resulted in deaths and many with long term illness (Fields, 2017; John et al., 2018; Rodriguez-Llanes et al., 2018). This is one of many examples of OPNA use in chemical warfare which suggests they may be used in the future (Okumura et al., 1996; Tucker, 1996; Yanagisawa et al., 2006; Jett, 2010; Nakagawa and Tu, 2018; Vale et al., 2018). OPNAs act as irreversible inhibitors of acetylcholinesterase which leads to accumulation of acetylcholine and induction of cholinergic crisis. The clinical signs following cholinergic crisis include salivation, lacrimation, urination, muscle weakness, respiratory dysfunction, and seizures (Millard et al., 1999; Jett, 2012; Ohbe et al., 2018; Richardson et al., 2019; Lott and Jones, 2020). Diisopropylfluorophosphate (DFP) is an organophosphate (OP) neurotoxicant commonly used to model the effects of historically used nerve agents such as soman and sarin (Lim et al., 1983; Deshpande et al., 2010; Todorovic et al., 2012; Flannery et al., 2016; Putra et al., 2019, 2020a).

Administration of a single high dose of DFP can lead to variable seizure activity and induction of *status epilepticus* (SE), a period of prolonged seizures (Jett, 2007; Putra et al., 2019; Rojas et al., 2019; Gage et al., 2020). SE can in turn lead to the development of epilepsy, the process known as epileptogenesis, which is characterized by spontaneous recurrent seizures (SRS) (Todorovic et al., 2012; Pitkänen and Engel, 2014; Tse et al., 2014; Seinfeld et al., 2016; Rojas et al., 2018; Putra et al., 2020a). DFP-induced *SE* can also lead to both short- and long-term changes such as gliosis, neurodegeneration and behavioral deficits (Deshpande et al., 2010; Guignet et al., 2020; Putra et al., 2020a). Currently, medical countermeasures (MCM) for cholinergic crisis include the administration of atropine sulfate (an anticholinergic), oximes (for reactivation of acetylcholinesterase) and benzodiazepines such as diazepam or midazolam (GABA agonists) (McDonough and Shih, 1997; Worek et al., 2010; Eddleston and Chowdhury, 2016). Delayed administration of MCMs, as is often the case in an in-hospital scenario, is often ineffective in mitigating the long-term impacts of OPNA exposure (Jett and Spriggs, 2018; Kuruba et al., 2018; Wu et al., 2018). Previous studies investigated the time course of benzodiazepine administration to mitigate DFP-induced effects (Todorovic et al., 2012; Kuruba et al., 2018; Wu et al., 2018). In one of these studies in rats, behavioral seizures were terminated and brain pathology was prevented when midazolam was administered 10-min after DFP exposure. However, midazolam was ineffective when it was administered 40, 60, and 120-min post-DFP (Wu et al., 2018). Another study showed a more immediate reduction in spike wave discharge frequency and amplitude when diazepam was given 10-min after DFP intoxication compared to animals given diazepam 30-min after DFP intoxication (Todorovic et al., 2012). The previous studies advanced our understanding of the effects of benzodiazepines timing on behavioral and neurological outcome in the rat DFP model but did not consider the initial severity of SE [i.e., the duration of convulsive seizures (CS)], induced by DFP. Generally, seizures can be ranked on a scale (modified Racine scale) where stages 1-2 are non-CS and stages 3-5 are CS (Racine, 1972). Therefore, our study aims to determine the effects of the duration of CS during SE i.e., between DFP exposure and the administration of midazolam, on gliosis and neurodegeneration.

Although the current MCMs do have desired efficacy in controlling the clinical signs when administered shortly after intoxication, there are no countermeasures or disease-modifying agents available to mitigate the long-term neurotoxicity. Our laboratory has recently tested the disease modifier, saracatinib (SAR or AZD0530) following exposure to repeated low doses of kainate (KA), resulting in SE (Sharma et al., 2018, 2021). SAR is an inhibitor of Src family tyrosine kinases (SFKs) which are implicated in several developmental, neuroinflammatory, and synaptic plasticity pathways (Ingraham et al., 1989; Boggon and Eck, 2004; Ohnishi et al., 2011; Nie et al., 2020; Gage and Thippeswamy, 2021). SFKs have been implicated in a variety of neurological diseases, including Parkinson’s and Alzheimer’s diseases (PD, AD), stroke, chronic pain and epilepsy (Lee et al., 2004; Kaufman et al., 2015; Knox and Jiang, 2015; Miyamoto et al., 2017; Sharma et al., 2018, 2021; Panicker et al., 2019; Ge et al., 2020; Yang et al., 2020). In neurons, phosphorylated Fyn, a SFK, interacts with tau in response to seizures and can lead to phosphorylation of N-methyl D-aspartate receptors (NMDAR) and contribute to glutamatergic toxicity (Nakazawa et al., 2001; Yang and Leonard, 2001; Ittner et al., 2010; Trepanier et al., 2012; Putra et al., 2020b). Phosphorylated Fyn can also regulate the expression of metabotropic glutamatergic receptors (Jin et al., 2017). SFKs also play a role in microglia. For example, c-Src was found to be necessary and sufficient for microglia activation both *in vitro* and *in vivo* (Socodato et al., 2015). Fyn phosphorylates protein kinase C delta (PKCδ) which leads to the translocation of nuclear factor-kappa (NFκB) to the nucleus and transcription of key proinflammatory cytokine genes and the production of reactive oxygen species in both PD and epilepsy models (Panicker et al., 2015, 2019; Sharma et al., 2018, 2021).

Saracatinib (SAR) has been tested in clinical trials for AD, though with limited efficacy, which could be due to delayed administration of the drug after the disease onset (Baselga et al., 2010; Fujisaka et al., 2013; Nygaard et al., 2014, 2015; Van Dyck et al., 2019). In our previous study using the rat KA model of epileptogenesis, SAR administration 2 hours following diazepam led to a significant reduction in SRS, epileptiform spike rate, gliosis, and neurodegeneration compared to the vehicle control (Sharma et al., 2021). As DFP induces SE like KA, we predicted that SAR administration may also mitigate neurodegeneration and gliosis in the DFP model. In this study, we tested the tolerability of SAR dosing regimens, which was administered 2 h post-midazolam, in ∼ 20-min and > 60-min of continuous CS groups. We also tested the effects of SAR on gliosis and neurodegeneration in rats with ∼20-min continuous CS during SE.

## Materials and Methods

### Animals, Care, and Ethics

Male Sprague Dawley rats (7-8 weeks) were purchased from Charles River (Wilmington, MA, United States). We used 81 animals in this study. Animals were randomly assigned to either DFP or vehicle (phosphate buffered saline, PBS) treatment. At the end of the study, all animals were euthanized using pentobarbital sodium (100 mg/kg, i.p.). Some animals were perfused for immunohistochemistry (IHC) while other animals were sacrificed for Western blotting (WB). The procedures were approved by the Institutional Care and Use Committee (IACUC-18-159) at the Iowa State University. Animals were single housed with alpha dri bedding and enrichment materials at the Iowa State Laboratory of Animal Resources and given *ab libitum* access to food and water with 12-h light/dark cycles.

### Chemicals

Diisopropylfluorophosphate was purchased from Sigma-Aldrich, stored at −20°C, and prepared in cold 0.1M PBS at desired concentration just prior to administration. Atropine sulfate (ATS, Thermo Fisher Scientific) and 2-pralidoxime (2-PAM, Sigma-Aldrich) were prepared fresh in saline at 5 mg/mL and 50 mg/mL, respectively. Midazolam (MDZ, prepared as 5 mg/mL stock solution) and pentobarbital sodium were purchased from the Iowa State University Lloyd Veterinary Medical Center Hospital Pharmacy.

Antibodies used for IHC and WB in this study include ionized calcium binding adaptor molecule (IBA1, goat, 1:500 for IHC and 1:1000 for WB, Abcam) for microglia, cluster of differentiation 68 (CD68, rabbit polyclonal, 1:400, Sigma) for phagocytic microglia, glial fibrillary acidic protein (GFAP, mouse monoclonal, 1:400 for IHC, 1:1000 for WB, Sigma) for astrocytes, and NeuN (rabbit, polyclonal, 1:400, Millipore) for neurons. B eta-actin (Sigma-Aldrich) was used to normalize Western blots (1:10,000). NeuN and Flouro-Jade B (FJB, Hisotchem) were used for the assessment of neurodegeneration. FITC conjugated (1:80), biotin conjugated (1:400), and streptavidin conjugated (1:300) antibodies were purchased from Jackson ImmunoResearch Laboratories. Antibodies were diluted in PBS containing 2.5% donkey serum, 0.1% tritonX-100, and 0.25% sodium azide. Streptavidin conjugated antibodies were diluted in PBS without a detergent. FJB was diluted in 0.1% acetic acid. Radioimmunoprecipitation assay (RIPA) buffer and protease and phosphatase (PPI) were purchased from Thermo Fisher Scientific. Blocking buffer was from Licor and antibodies for Western blotting were diluted in 1:1 blocking buffer and PBS with 0.1% tween 20.

Saracatinib (SAR/AZD0530) was supplied by AstraZeneca through the Open Innovation program. SAR was prepared 6 mg/kg in vehicle containing 0.5% hydroxypropyl methylcellulose and 0.1% tween 80. The vehicle was prepared by first heating DNAase-free sterile water to 90°C before adding hydroxypropyl methylcellulose. Solution was then cooled to room temperature before the addition of tween 80. Vehicle was then autoclaved and cooled to room temperature before adding finely grounded SAR. SAR is left stirring overnight, in the dark, before dosing the next day. SAR was left stirring at room temperature throughout the dosing period to avoid precipitation.

### Induction of *Status Epilepticus* by Diisopropylfluorophosphate, Classification of Animals by Status Epilepticus Severity and Duration, and Saracatinib Treatment

Four milligram/kg DFP was administered (s.c.) to rats followed immediately by 2 mg/kg (ATS, i.m.) and 25 mg/kg 2-PAM (i.m.) to reduce mortality. Within 5-10-min following DFP administration, animals typically developed seizures which were assessed in real time using a staging scale as previously described (Gage et al., 2020; Putra et al., 2020a). Stages 1-2 were considered non-convulsive seizures (NCS) while stages 3-5 were considered convulsive seizures (CS). Stage one was characterized by salivation, lacrimation, urination, defecation (SLUD) and repetitive mastication. Stage two was characterized by head nodding, tremors, and a hunched posture. Stage three was characterized by rearing, Straub tail, and bicycling of the forearms. Stage four included forelimb clonus and the loss of righting reflex, while stage five included abducted limbs or repeated rearing and falling, and/or jumping. Each minute was assigned a score for each animal based on the stage of seizure. Midazolam (3 mg/kg, i.m.) was administered 30-min, 60-min, or 120-min post-DFP (i.e., ranging from 30-min to 2 h post-DFP).

**FIGURE 1:**
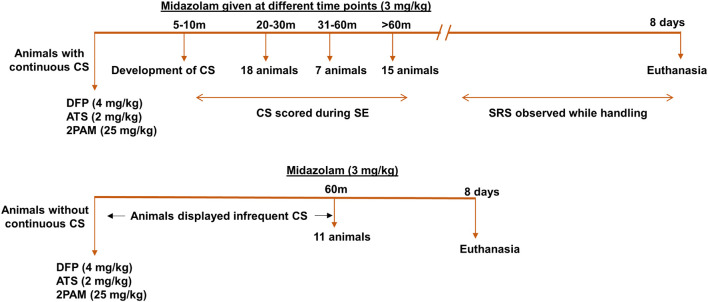
Experimental design. Animals were administered 4 mg/kg DFP (s.c.) immediately followed by 2 mg/kg ATS (i.m.) and 25 mg/kg 2-PAM (i.m.) and either developed continuous convulsive seizures (stage 3-5) or no continuous convulsive seizures. Animals were administered midazolam after animals had 20-min convulsive seizures (around 30-min post DFP), one hour or two hours post DFP to generate various durations of convulsive seizures (CS). Animals without continuous convulsive seizures were given midazolam one hour after DFP. Animals were terminated 8 days post DFP. CS, convulsive seizure, DFP, diisopropylfluorophosphate, ATS, atropine sulfate, 2-PAM, 2-pyridoxime.

**FIGURE 2:**
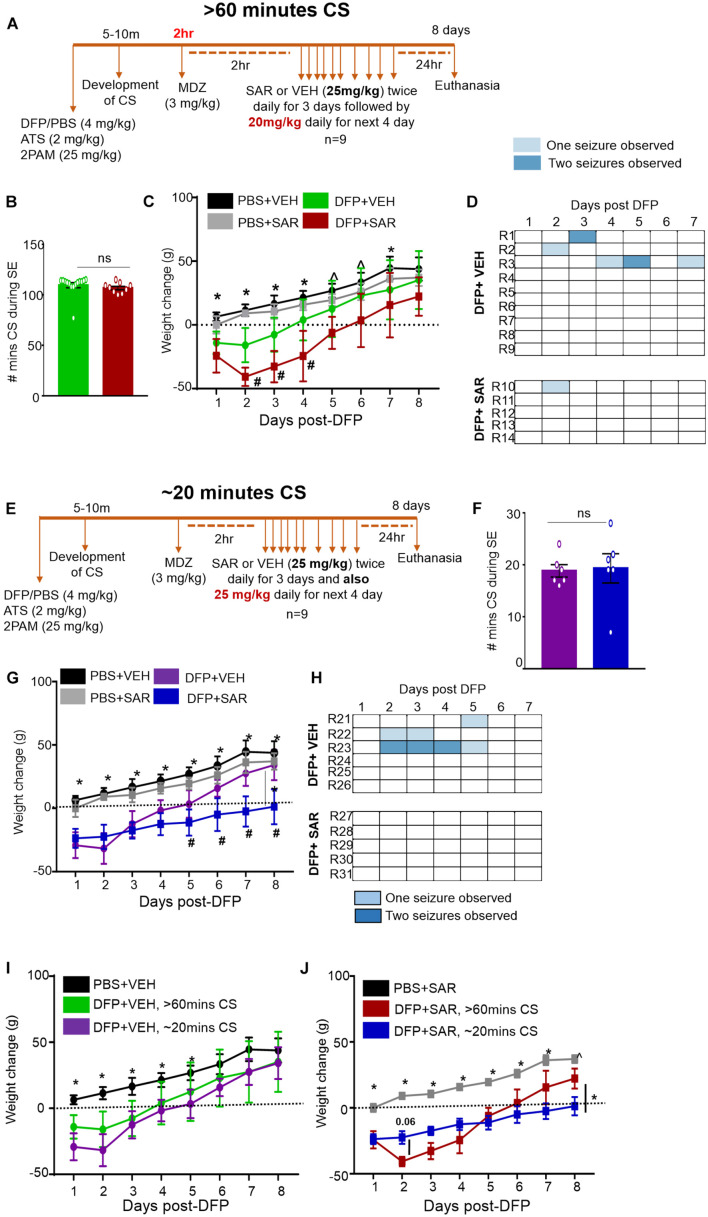
Experimental timelines, SE severity, bodyweight and observed spontaneous seizures mitigation by saracatinib (SAR) during 7 days post-DFP intoxication. **(A)** Experimental timeline for animals with > 60-min CS. **(B)** There was no significant difference between the number of minutes of CS during SE between vehicle and SAR treated groups (*t*-test, *n* = 9-15). **(C)** Change in bodyweight over the 7 days post DFP (mixed measures ANOVA, *n* = 9-14). **(D)** Heat-map representation of the number of SRS per day. **(E)** Experimental timeline for animals with ∼20-min CS. **(F)** There was no significant difference between the number of minutes of CS during SE between vehicle and SAR treated groups (*t*-test, *n* = 6). **(G)** Change in bodyweight over the 7 days post DFP (mixed measures ANOVA, *n* = 5-6). **(H)** Heat-map representation of the number of SRS per day. **(I,J)** Change in bodyweight comparisons between > 60-min and ∼20-min CS groups in DFP + VEH and DFP + SAR groups (mixed measures ANOVA, *n* = 5-15). *p < 0.05 both DFP compared to controls, ^*p* < 0.05 DFP + SAR group compared to controls, #*p* < 0.05 DFP + VEH compared to DFP + SAR group. Error bars indicate standard error of the mean.

The experimental designs are illustrated in Figures 1, 2A,E. 10 animals were administered PBS as controls for DFP; these animals are termed “controls” here on. 5 animals were administered PBS and SAR as control for DFP + SAR group to investigate the effects of SAR on its own. We exposed 66 animals to DFP for this study. Following DFP exposure, 11 animals had non-continuous CS (< 20 min); this group is classified as the “no continuous SE” (NCSE) group. The remaining 55 DFP administered animals developed CS within 5-10-min and had continuous CS activity. To achieve different durations of continuous CS, we administered midazolam at various time points. These animals were categorized as having either ∼20-min continuous CS (*n* = 24) 31-60-min continuous CS (*n* = 7), or > 60-min continuous CS (*n* = 24). From these DFP treated groups, SE severity matched animals were chosen randomly from the ∼20-min group (*n* = 6) and the > 60-min group (*n* = 9) for SAR or vehicle administration, which started two hours post-midazolam. Based on our previous study in the rat KA model (Sharma et al., 2021), in both ∼20-min and > 60-min groups, animals were given 25 mg/kg SAR (o.p.) twice a day for the first three days. Considering the SE severity, animals with > 60-min CS were administered 20 mg/kg SAR (o.p.) once a day for the next four days while animals with ∼20-min CS were administered 25 mg/kg once a day for the next four days. In addition to the 66 animals previously mentioned, in a separate study, we had also administered diazepam to 20 animals 2 h following DFP without any countermeasures. These animals were reported in our previous study (Putra et al., 2019). However, in this study, we only used the mortality data solely to compare the effects of diazepam and midazolam in mitigating mortality in DFP model. All animals were euthanized and perfuse-fixed with 4% paraformaldehyde 24 h after the last dose of SAR or vehicle (i.e., 8 days post DFP exposure).

### Evaluation of Spontaneous Recurrent Seizures While Handling

Electroencephalography (EEG) is the most robust method for the evaluation of seizures as it allows for continuous recording and detection of both convulsive and non-convulsive seizures (Bassett et al., 2014; Puttachary et al., 2015; Papazoglou et al., 2016; Sharma et al., 2021). Although the animals were not telemetered in this short-term drug-tolerability study, we knew that animals were likely to have spontaneous CS when handled by the experimenter or when the cages were cleaned by an animal attendant. Animals were handled by the experimenter twice daily (once in the morning and once in the evening), as a routine husbandry practice or while dosing, and the number of spontaneous seizures was recorded throughout the seven-day period. Notably, we did not consider seizures during the first 24 h after DFP exposure as SRS due to the potential of DFP remaining in circulation and recovery time for acetylcholinesterase (Gearhart et al., 1994; Chen et al., 2009). Spontaneous CS were characterized by rearing, Straub tail, bicycling of limbs, closed eyes, and sometimes excessive jumping.

### Immunohistochemistry

Animals were perfused (60 mL/min at 80 mm Hg) with PBS followed by 4% paraformaldehyde (PFA). Brains were dissected and incubated in 4% PFA before being transferred to 25% sucrose in PBS for at least 48 h. Brains were then gelatin embedded (15% type A porcine gelatin, 7.5% sucrose, 0.1% sodium azide) overnight at 4°C before freezing in liquid nitrogen, cooled by isopentane. Brains were then sectioned (16 μm) using a cryostat and collected onto gelatin coated slides so that each slide contained sections of the hippocampus, piriform cortex, and amygdala from rostral to caudal as described in our previous publication (Puttachary et al., 2016). Slides were stored at −20°C prior to staining.

The brain sections were treated with citric acid solution (10 mM citric acid and 0.05% tween-20, pH 6) at 95°C for 23 min for antigen retrieval. After cooling for 30-min, slides were processed for IHC. Slides were placed into Shandon racks and washed with PBS for 1 h. Slides were then incubated for one hour in blocking buffer (10% donkey serum, 0.05% TritonX-100 in PBS) before incubating with primary antibodies overnight at 4°C. The following day, slides were washed for one hour in PBS and incubated with FITC conjugated or biotinylated conjugated secondary antibodies for one hour. Slides were washed again for one hour with PBS before incubating with streptavidin conjugated antibodies for one hour followed by washing with PBS for one hour. Slides were then mounted with medium containing DAPI and used for imaging. For FJB staining, following staining with NeuN and washing with PBS, slides were washed 3-4 times with distilled water before being placed in 0.006% potassium permanganate for 10-minutes (Todorovic et al., 2012). Slides were again washed in distilled water 3-4 times before being submerged in 0.0003% FJB solution. FJB stained slides were dried and dipped in xylene for clearing before applying Surgipath acrytol. After staining, slides were stored at 4°C until imaging.

The Axiovert 200 Zeiss inverted fluorescence microscope with Hamamatsu camera was used to image the slides at various brain regions (20X). The regions included the dentate gyrus (DG), cornu ammonis 3 (CA3), two regions of cornu ammonis 1 (CA1, one in the middle of CA1 and one close to the subiculum), the piriform cortex (PC) and amygdala (AMY). To compare with WB findings, we also pooled the IHC data from all hippocampal regions and then separately pooled IHC data from the PC and AMY. Based on previous findings in the DFP model, these regions were the most affected brain regions for gliosis and neurodegeneration and (Aroniadou-Anderjaska et al., 2009, 2016; Flannery et al., 2016; Hobson et al., 2018; Putra et al., 2019).

Epilepsy, regardless of the model, is characterized by increases in glial cell numbers as well as morphological changes. For IHC, a minimum of four sections per animal were used for evaluation per staining. To evaluate microgliosis, slides were stained with IBA1 and CD68. The total number of microglia and the total number of microglia containing CD68 were counted. IBA1 positive cells were also evaluated for morphology. Cells with long processes and small cell bodies were considered non-reactive (M2-like) microglia while cells with short processes and large cell bodies were considered reactive microglia (M1-like) (Streit et al., 1999). Similarly, astrogliosis was evaluated using GFAP. The total number of GFAP positive cells was considered. Astrocytes were also evaluated for morphology. The cells with small cell bodies and extended processes were considered non-reactive (A2-like) astrocytes and cells with large cells bodies and retracted processes were considered reactive (A1-like) astrocytes (Li et al., 2019). Image J was used to manually count and evaluate glial cells for morphology. Notably, glial morphology exists on a spectrum and manual counting is subject to bias without specific automated criteria for process length and cell body size (del Fernández-Arjona et al., 2017; Zhou et al., 2019). To evaluate neurodegeneration, we counted the number of FJB positive cells (manually) and the number of NeuN positive cells (using an automatic cell counting software, Cell Profiler). Cell profile pipeline included a 22-120 pixel range, with a global, Otsu thresholding strategy. Experimenters were blind to the treatment groups while counting.

### Western Blotting

Following euthanasia, hippocampal and PC-AMY tissues were dissected from the brain and one hemisphere was used for Western blotting analysis. We used tissues from the controls, ∼20 min group and > 60-min group. Tissues were snap frozen in liquid nitrogen and stored at −80°C prior to processing. Tissues were homogenized (1 μg/1 μl) in RIPA buffer containing 1% protease and phosphatase inhibitor. Sonication was used to further homogenize samples before centrifugation at 10 RCF for 1 h. The supernatant was collected, aliquoted and stored at −80°C. 30 μg of protein from each sample were loaded into each well of an SDS–PAGE gel (10–15%). 2 μl of molecular weight marker (Bio-Rad) was loaded to determine protein sizes. Gels were run at 4°C for 1-2 h at 110 mV. Proteins were then transferred overnight (4°C) onto a nitrocellulose membrane. Blots were then washed with PBS prior to a one-hour incubation with blocking buffer. Membranes were incubated at 4°C for ∼16 h in primary antibodies. Membranes were then washed with PBS- T (PBS containing 0.1% tween 20) followed by incubation with IR-680 or IR-800 dyes conjugated to secondary species antibodies. After washing again with PBS-T, membranes were then incubated in a similar method with primary β-actin antibody as a loading control. Appropriate IR-680 or IR -800 dyes were used to detect β-actin. The bands were visualized with Odyssey IR imaging system and proteins were identified by molecular weight using the standards. Image Studio Lite was used to quantify the bands which were normalized to β-actin.

### Statistical Analysis

Graphpad Prism 7.0 was used to analyze data and make graphical presentations. Normality was assayed using the Shapiro–Wilk test. The Grubbs’ test was used to eliminate outliers. One-way ANOVA was used unless data was considered not normal in which the Kruskal Wallis test was performed. In cases in which another independent variable is introduced (ex. brain region), mixed measures ANOVAs were utilized. Specific statistical analysis is further indicated in the corresponding figure legends.

## Results

### Severity and Duration of Status Epilepticus and Its Impact on Bodyweight and Mortality

**FIGURE 3:**
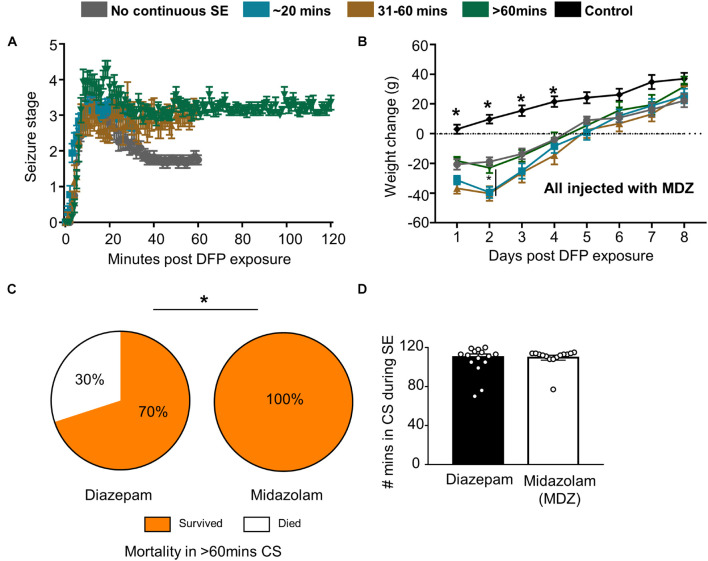
Impact of SE duration and severity on bodyweight and mortality. **(A)** Following DFP, animals were monitored for seizure severity using a modified Racine scale (1-5) on each minute. Average seizure stage at each minute is depicted. **(B)** Weight change over time for each group of animals (mixed measures ANOVA, *n* = 7-15). **(C)** When midazolam was given 2 h following DFP, surprisingly no mortality was observed (in the first 24 h compared to animals treated with diazepam from a previous study (Chi-square, *n* = 15-20). **(D)** There was no significant difference in seizure severity (number of minutes of convulsive seizures) between the animals given midazolam and diazepam (*t*-test, *n* = 15-20). Error bar indicates standard error of the mean. *p < 0.05 compared to DFP groups or as indicated by the vertical lines.

Following DFP intoxication, animals developed both NCS and CS. Animals were divided into groups based on the number of minutes spent in CS (Figures 1, 3A). Most animals had continuous CS during SE but in a subset of animals (11 out of 66) CS did not persist (NCSE group). Bodyweight was measured each day at the same time to compare recovery among the groups. Animals that died during the post-treatment or were treated with SAR were excluded from the analysis. Regardless of SE severity and duration or MDZ administration, all DFP exposed groups lost weight compared to PBS controls but recovered by day 5 (Figure 3B). The weight change for all DFP groups was statistically different for the first four days following DFP intoxication compared to controls. Interestingly, the NCSE and > 60-min groups recovered faster with significant increases in weight change at day two compared to the other DFP treated groups (Figure 3B). Although physical characteristics were not systematically quantified, several animals in all DFP treated groups exhibited changes consistent with the modified Irwin scale for morbidity (Irwin, 1968; Rojas et al., 2016). These characteristics included exophthalmos, tremors, muscle weakness, aggressive behavior, lacrimation, piloerection, and hunched posture.

Despite the morbidity, surprisingly, there was no mortality in all the DFP exposed and midazolam treated groups that had > 20-min of continuous CS. However, 2 out of 11 animals died in < 6 days in the NCSE group despite treating with midazolam. In our previous DFP studies with diazepam, we observed increased mortality in a group that had > 60-min of CS (Putra et al., 2019). When compared with the > 60-min group in this study, midazolam significantly reduced mortality compared to diazepam (Figure 3C). In the diazepam treated groups, 30% mortality occurred in < 24 h of DFP exposure (Putra et al., 2019). Importantly, there was no difference in SE severity between the midazolam and diazepam treated groups (Figure 3D).

### The Impact of Status Epilepticus Severity and Duration on the Occurrence of Spontaneous Seizures Observed While Handling

**FIGURE 4:**
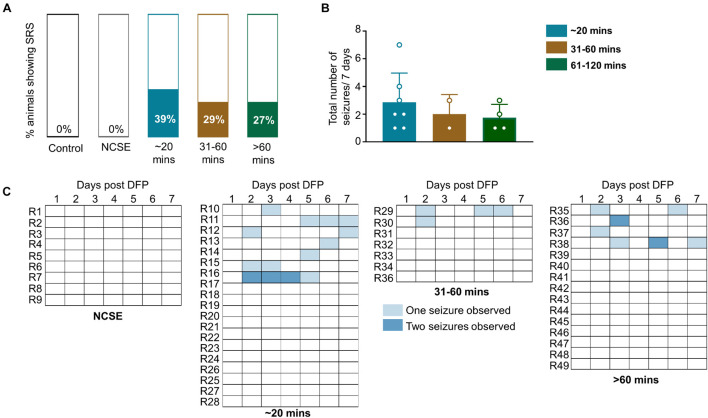
Impact of SE duration and severity on observed spontaneous convulsive seizures while handling or soon after handling. Animals were handled twice a day for a week post-DFP. **(A)** 39% of animals that had 20-30-mins CS, 29% of animals with 31-60-min CS and 27% animals with > 60-min CS had spontaneous convulsive seizures. **(B)** There was no significant difference between the total numbers of seizures for the 7-day period between the groups (Kruskal Wallis, *n* = 2-4). **(C)** Representation of frequency of seizures over the 7-day period. NCSE, no continuous CS during SE. Error bars, standard error of the mean.

Animals were handled twice a day for a week. Following or during handling, some animals displayed a spontaneous (not induced by chemoconvulsant) CS (SRS). This was recorded across the groups. Approximately, one third of the animals (27–39%) in all DFP treated groups, except the NCSE group, displayed at least one spontaneous CS over the seven-day period post-DFP (Figure 4A) suggesting their epileptic state. However, there were no statistical differences between the groups for the total number of seizures experienced over the seven-day period (Figure 4B). The SRS observed for each day is presented in a heatmap (Figure 4C).

### The Impact of Status Epilepticus Severity and Duration on Gliosis and Neurodegeneration

Gliosis and neurodegeneration are well known hallmarks of epileptogenesis. We utilized IHC with cell-specific markers to observe both glial cells and neurons in the hippocampus (two regions of CA1- one close to the subiculum and the other in the middle of CA1, CA3, and DG) and extra-hippocampal regions (PC and AMY). Interestingly, the parameters of interest were usually consistently upregulated in the PC and AMY at 8-day post-DFP in contrast to the regions of the hippocampus. Therefore, we used mixed measures ANOVAs in the hippocampal and extra-hippocampal regions separately (*n* = 5-9). Each DFP treated group was compared to the control (PBS) group. To compare with WB data, we pooled the IHC data from all hippocampal regions as well as the data from the PC and AMY. Western blotting was conducted for IBA1 and GFAP to further analyze gliosis. Notably, we only used Western blotting in the controls, ∼20-min group and > 60-min group as there were minimal differences between the controls and NCSE group as well as between the 31-60-min group and > 60-min group.

#### The Impact of Status Epilepticus Severity and Duration on Microgliosis

**FIGURE 5:**
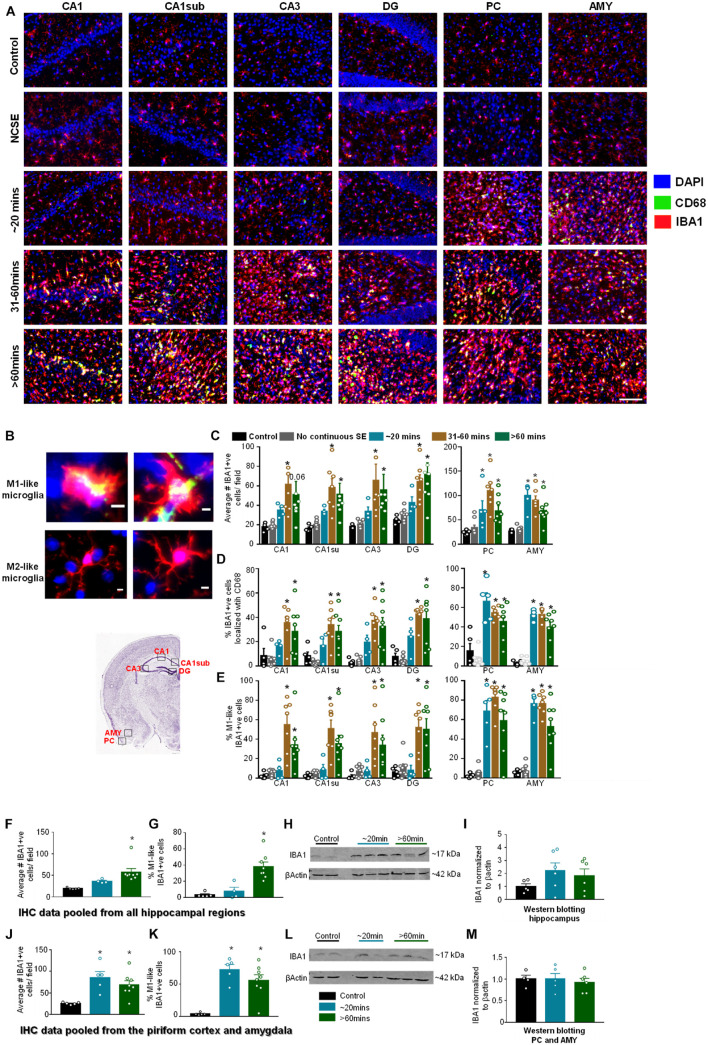
Impact of SE severity and duration on microgliosis. **(A)** Representative images of the hippocampus (CA1, CA1 close to the subiculum = CA1sub, CA3, DG), piriform cortex (PC) and amygdala (AMY). **(B)** Examples of reactive (M1-like, large cell body with retracted processes) and non-reactive (M2-like, small cell body with numerous fine branching patterns) IBA1 positive cells. M1-like microglia usually contained CD68 (yellow in the top panels). **(C)** Average number of IBA1 positive cells, **(D)** percent IBA1 positive cells colocalized with CD68, **(E)** percent IBA1 positive cells with reactive (M1)-like morphology (mixed measures ANOVA, *n* = 5-9). **(F,G)** Pooled data from all the hippocampal regions for the numbers of IBA1 positive cells **(F)** and IBA1 positive cells with reactive morphology (**G**, ANOVA, *n* = 5-8). **(H,I)** Western blotting for hippocampal lysates for IBA1 (ANOVA, *n* = 4-6). **(J,K)** Pooled data from the amygdala/piriform cortex for number of IBA1 positive cells **(J)** and IBA1 positive cells with reactive morphology (ANOVA, *n* = 5-8). **(L,M)** Western blotting for PC/AMY lysates for IBA1 (ANOVA, *n* = 4-6). Error bars indicate standard error of the mean. Scale, 100 μm **(A)** and 5 μm **(B)**. *p < 0.05 compared to control. All graphs- mixed effects ANOVA, *n* = 5-12. Area counted = 0.14 mm^2^. Atlas Image credit: Allen Institute, United States.

We investigated microgliosis using IBA1 and CD68 IHC (Figures 5A,B) to assess three parameters: the number of IBA1 positive cells (Figure 5C), the number of IBA1 positive cells co-labeled with CD68 (Figure 5D), and the percentage of IBA1 positive cells with reactive morphology (Figure 5E). To assess the morphology of IBA1 positive cells, reactive cells (M1-like) were considered to have large cell bodies and retracted processes and non-reactive microglia (M2-like) were considered to have small cell bodies and extended processes (Figure 5B). Representative images for each group at each location are shown in Figure 5A. Interestingly, in all hippocampal regions, all parameters were significantly upregulated in the 31-60-min and > 60-min group, but not in the ∼20-min group, compared to controls (Figures 5C–E). There was no increase for any parameter in the NCSE group in any region, and a non-significant increase was observed in the ∼20-min group for the total number of microglia and the percent of microglia colocalized with CD68. In the PC and AMY, there was a significant increase for all DFP treated groups (except for the NCSE group) for all three microgliosis parameters (Figures 5C–E).

Pooled IHC data from the hippocampus showed a significant increase in the number of IBA1 positive (Figure 5F) and percent of IBA1 positive cells with M1-like morphology (Figure 5G) in the > 60-minutes group compared to controls. In the Western blotting, compared to controls, there was an upregulation of IBA1 in the hippocampus for the ∼20-min group and > 60-min group though the differences were not significant (Figures 5H,I). In the pooled data from the PC and AMY, there was a significant increase in IBA1 positive cells (Figure 5J) and increase in the percentage of cells with M1-like morphology (Figure 5K) in both the ∼20-minutes and > 60-minutes groups compared to controls. However, Western blotting did not reveal any change in IBA1 levels between groups (Figures 5L,M).

#### The Impact of Status Epilepticus Severity and Duration on Astrogliosis

**FIGURE 6:**
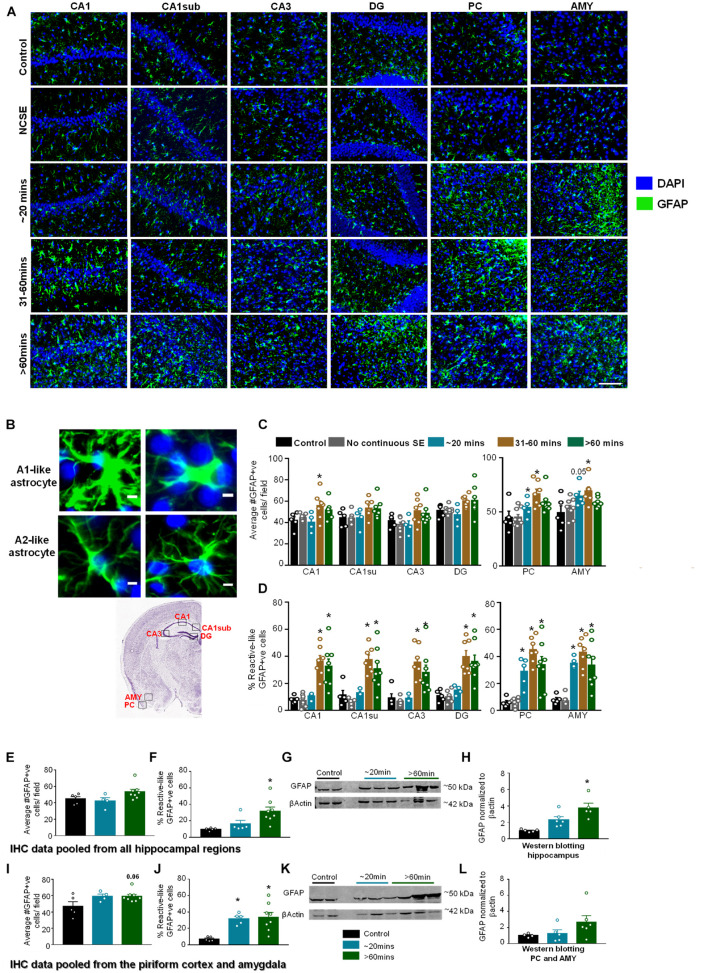
Impact of SE severity and duration on astrogliosis. **(A)** Representative images of the hippocampus (CA1, CA1 close to the subiculum = CA1sub, CA3, DG), piriform cortex (PC) and amygdala (AMY). **(B)** Examples of reactive (A1-like, large cell bodies, short and thick processes) and non-reactive (A2-like) GFAP positive cells. **(C)** Average number of GFAP positive cells, and **(D)** percent GFAP positive cells with reactive (A1) like morphology (mixed measures ANOVA, *n* = 5-9). **(E,F)** Pooled data from the hippocampus showing the number of GFAP positive cells **(E)** and GFAP positive cells with reactive morphology **(F)**. **(G,H)** Western blotting of hippocampal lysates for GFAP (ANOVA, *n* = 4-6). **(I,J)** Pooled data from the amygdala/piriform cortex for number of GFAP positive cells **(I)** and GFAP positive cells with reactive morphology (**J**, ANOVA, *n* = 5-8). **(K,L)** Western blotting of PC/AMY lysates for GFAP (ANOVA, *n* = 4-6). Error bars indicate standard error of the mean. Scale, 100 μm **(A)** and 5 μm **(B)**. *p < 0.05 compared to control. Both graphs- mixed effects ANOVA, *n* = 5-12. Area counted = 0.14 mm^2^ Atlas Image credit: Allen Institute, United States.

Glial Fibrillary Acidic Protein IHC was used to assess the morphology and the number of GFAP positive cells (Figures 6A–D). In this analysis, cells with large cell bodies and retracted processes were considered to be reactive astrocytes (A1-like) while cells with small cell bodies and extended processes were considered to be non-reactive (A2-like). Representative images are shown in Figures 6A,B. In hippocampal regions, compared to controls, there was an increase in GFAP positive cells in all regions for the 31–60-min group and > 60-min group though it was only significant for the 31–60-min group in CA1 (Figure 6C). For GFAP positive cells with reactive morphology, there was a significant upregulation in the 31-60-minutes group and > 60-min group for all regions of the hippocampus (Figure 6D). In the PC and AMY, there was an increase in GFAP positive cells and the percent of GFAP positive cells with reactive morphology in groups with ∼20-min CS (Figures 6C,D). In the PC and AMY, animals with ∼20-min CS had a trending increase in the number of GFAP positive cells in the PC and AMY and a significant increase in the percent reactive cells (Figure 6D).

Pooled IHC data from all regions of the hippocampus showed an increase in the number of GFAP positive cells (Figure 6E, not significant) and percentage of GFAP positive cells with reactive-like morphology (Figure 6F, significant) in the > 60-min group compared to controls. As revealed by Western blotting, compared to controls, there was an upregulation of GFAP in the hippocampus for the ∼20-min group and > 60-min group though the differences were only significant for the > 60-min group (Figures 6G,H). In the pooled data from the PC/AMY, there was a trending increase in GFAP positive cells for the > 60-min animals compared to controls (Figure 6I). There was increase in the percentage of cells with reactive-like morphology for the ∼20-min group and > 60-min group compared to controls (Figure 6J). After western blotting, the > 60-min group had an increase in GFAP compared to controls though the difference was not significant (Figures 6K,L).

#### The Impact of Status Epilepticus Severity and Duration on Neurodegeneration

**FIGURE 7:**
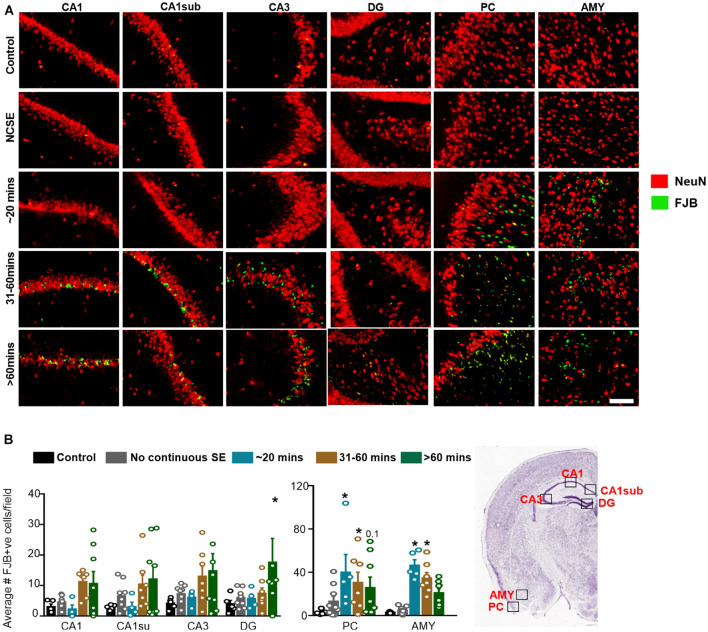
Impact of SE severity and duration on neurodegeneration. **(A)** Representative images of the hippocampus (CA1, CA1 close to the subiculum = CA1sub, CA3, DG), piriform cortex (PC) and amygdala (AMY). **(B)** Average number of FJB positive cells (mixed effects ANOVA, *n* = 5-9). Error bars indicate standard error of the mean. *p < 0.05 compared to control. Field = 0.14 mm^2^. Atlas Image credit: Allen Institute, United States.

To assess neurodegeneration, we utilized FJB staining co-labeled with NeuN. Interestingly, some FJB stain colocalized with NeuN positive cells while other FJB positive cells did not show NeuN positivity, which could be due to the downregulation of NeuN in neurons in response to DFP. Representative images are shown in Figure 7A. In the hippocampus, compared to controls, there was a non-significant upregulation of FJB positive cells in the 31-60-min group, and > 60-min group for all regions (Figure 7B). The only significant upregulation was in the > 60-min group in the DG. In the AMY and PC, compared to controls, there was an upregulation of FJB positive cells in the ∼20-min, 31-60-min, and > 60-min groups (Figure 7B). This was only significant for the ∼20-min and 31-60-min groups. There was no significant upregulation of FJB positive cells in the NCSE group.

### The Effects of Saracatinib Dosing Regimen in DFP-Induced Status Epilepticus Severity Models

Saracatinib is an inhibitor of Src family tyrosine kinases. We have previously reported that SAR treatment (25 mg/kg, twice daily for the first three days followed by a single dose per day for the next four days) during an early period of epileptogenesis in the rat KA model significantly reduced the number of SRS, gliosis, and neurodegeneration (Sharma et al., 2018, 2021). Since this dosing regimen was well tolerated in the KA model, we tested a similar dosing regimen, in the DFP model with some modification. We used two extreme SE severity groups; > 60-min and ∼20-min continuous CS groups. In both groups, the first three days of SAR dosing was the same as in the KA model i.e., 25 mg/kg, twice a day, but the dosing for the next four days was different. In the > 60-min group, we administered 20 mg/kg once a day for the next four days while in the ∼20-min CS group, we maintained 25 mg/kg single dose/day for the next four days. We decided to reduce the dose by 5 mg/kg in the > 60-min group due to the dramatic weight loss and mortality compared to the ∼20 minutes group (see section “Saracatinib Effects on Bodyweight, Mortality, and Spontaneous Recurrent Seizures Mitigation During the Treatment Period”). The experimental designs are depicted in Figures 2A,E. In both groups, to test SAR tolerability, we considered bodyweight, mortality, and SRS occurrence while handling. In the latter group, we further analyzed the effects of SAR on epileptogenic markers such as gliosis and neurodegeneration.

#### Saracatinib Effects on Bodyweight, Mortality, and Spontaneous Recurrent Seizures Mitigation During the Treatment Period

Animals were administered DFP and MCM followed by midazolam either 2 h or ∼30-min after intoxication (Figures 2A,E). During this period, the animals had > 60-min or ∼20-min of continuous CS (Figures 2B,F). Two hours later animals were administered either vehicle (VEH) or SAR. Importantly, there were no differences in number of minutes the animals spent in CS (VEH vs SAR) for either treatment regimen (Figures 2B,F). There was no difference in bodyweight change between the PBS + VEH and PBS + SAR treated animals (Figures 2C,G) suggesting SAR on its own was well tolerated in healthy adult rats. All DFP treated animals, regardless of VEH or SAR treatment lost bodyweight during the first 4 days. On most days there was a significant difference in weight change between the PBS and DFP treated groups (Figures 2C,F). In both > 60-min and ∼20-min CS groups, the DFP + VEH animals regained their bodyweight to their baseline level by 4-5 days post-DFP. While the SAR treated animals took 6-8 days (Figures 2I,J). In the animals with > 60-min CS, DFP + SAR treated animals that received 20 mg/kg daily in the last four days (after completing twice daily doses of 25 mg/kg in the first three days) gained weight slower than DFP + VEH treated animals though the difference was not significant (Figure 2C). In the ∼20-min CS group, DFP + SAR animals that received 25 mg/kg throughout had a significant reduction in weight change compared to DFP + VEH between days 5 and 8 (Figure 2G). Surprisingly, in the DFP + SAR treated animals in > 60-min CS group, 4 out of 9 animals died during treatment despite reducing the dose by 5 mg/kg after 3 days. In the ∼20-min CS group, only 1 out of 6 animals died during the treatment period. Despite mortality in SAR-treated groups, none of the survived animals in the ∼20-min CS group, and one animal in the > 60-min CS group, had SRS. In contrast, 46% of the animals in the vehicle-treated groups had SRS during the first week of post-DFP (Figures 2D,H)-A heat map of SRS during the treatment period is represented in Figures 2D,H.

#### Saracatinib Reduces Diisopropylfluorophosphate Induced Gliosis and Neurodegeneration

**FIGURE 8:**
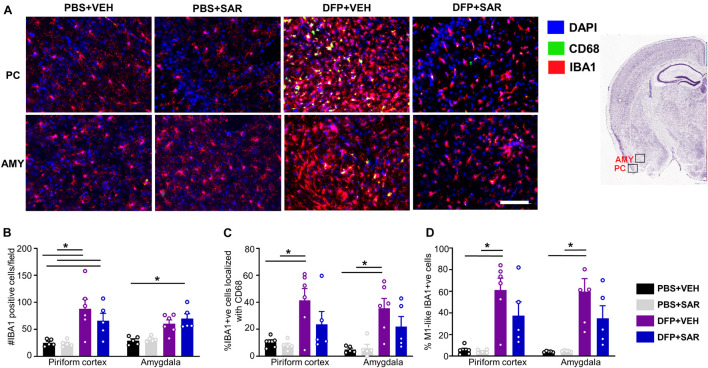
Effects of SAR on DFP-induced microgliosis. **(A)** Representative images from the piriform cortex (PC) and amygdala (AMY) showing IBA1 and CD68 positive cells. **(B,D)** DFP induced significant upregulation of IBA1 positive cells **(B)**, IBA1 positive cells colocalized with Cd68 **(C)** and IBA1 positive cells with reactive (M1) like morphology **(D)** with non-significant mitigation by SAR (mixed measures ANOVA, *n* = 5-6). Mixed measures ANOVA, *n* = 5-6. Error bars indicate standard error of the mean. *p < 0.05. Area counted = 0.14 mm^2^ Atlas Image credit: Allen Institute, United States.

Due to increased mortality in DFP + SAR treated animals with > 60- min CS, we focused our analysis to animals with ∼20-min CS. We utilized glial and neuronal marker IHC to determine the efficacy of SAR in reducing gliosis and neurodegeneration. As animals with ∼20-min of continuous SE did not have significant pathology in the hippocampus, we focused the analysis to the PC and AMY. Representative images of microglia (IBA1 positive) and CD68 from each treatment group are presented in Figure 8A. Compared to controls, DFP led to a significant increase in the number of IBA1 positive cells, the percent of IBA1 positive cells colocalized with CD68, and the percent of IBA1 positive cells with M1-like morphology in both PC and AMY (Figures 8B–D). Compared to VEH treated animals SAR-treated group had a reduction of IBA1 positive cells with CD68 (43% in PC, 38% in AMY) and M1-like microglia (38% in PC, 41% in AMY). However, the differences were not significant (Figures 8C,D).

**FIGURE 9:**
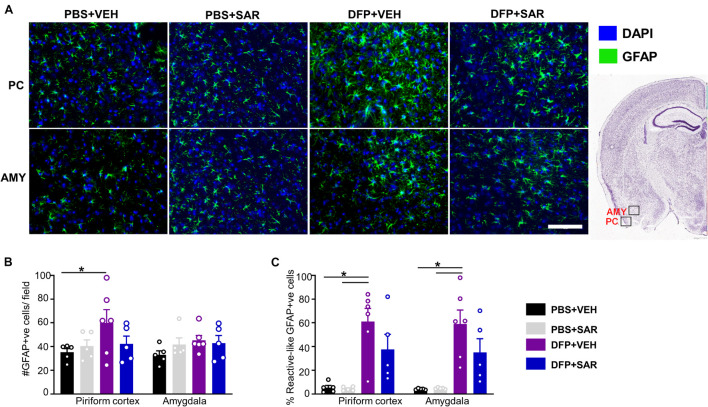
Effects of SAR on DFP-induced astrogliosis. **(A)** Representative images of GFAP positive images from the piriform cortex (PC) and amygdala (AMY). **(B,C)** DFP led to a significant upregulation of GFAP positive cells **(B)** and GFAP positive cells with reactive (A1) like morphology **(C)** with significant modulation by SAR in the AMY, however; the difference in the PC was not significant (mixed measures ANOVA, (*n* = 5-6). GFAP = glial fibrillary acidic protein, DAPI = 4′,6-diamidino-2-phenylindole. Error bars indicate standard error of the mean. Mixed measures ANOVA, *n* = 5-6. *p < 0.05. Area counted = 0.14 mm^2^ Atlas Image credit: Allen Institute, United States.

Representative images of astrocytes (GFAP positive) are shown in Figure 9A. In the PC, there was a significant increase in total GFAP positive cells in the DFP + VEH group compared to controls and a non-significant reduction (30%) by SAR (Figure 9B). In both the PC and AMY, there was a significant increase in GFAP positive cells with A1-like morphology compared to controls with non-significant reduction (39% in PC, 40% in AMY) by SAR (Figure 9C).

**FIGURE 10:**
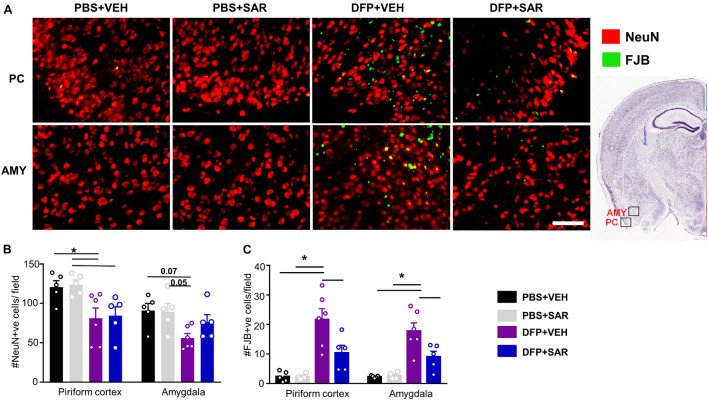
Saracatinib (SAR) mitigated DFP-induced neurodegeneration in AMY. **(A)** Representative images of NeuN + FJB staining from PC and AMY are shown. **(B)** DFP, regardless of treatment with vehicle or SAR, led to a significant reduction in NeuN positive cells in the PC. However, in the AMY the changes were not significant though there was an increase of NeuN positive cells in SAR compared to VEH (mixed measures ANOVA, *n* = 5-6). **(C)** FJB staining revealed a significant increase of degenerating neurons in DFP + VEH and significant mitigation by SAR (mixed measures ANOVA, *n* = 5-6). Error bars indicate standard error of the mean. Mixed measures ANOVA, *n* = 4-6. *p < 0.05. Field = 0.14 mm^2^ Atlas Image credit: Allen Institute, United States.

Neurodegeneration was assessed by counting the total number of NeuN positive cells and the number of FJB positive cells. Representative images are shown in Figure 10A. There was a significant reduction in NeuN positive cells by DFP, compared to controls, regardless of treatment in the PC (Figure 10B). In the AMY there was a reduction in NeuN positive cells by DFP and some mitigation (35%) by SAR though these differences were not significant. In both the PC and AMY, there was a significant increase in FJB positive cells in the DFP + VEH animals compared to controls and a significant reduction by SAR (Figures 10A,C).

## Discussion

The purposes of this study were to determine the impact of SE severity and duration on epileptogenic parameters and to test the disease-modifying potential of the Src family tyrosine kinase inhibitor, SAR. Currently, OP intoxication-induced SE is combated by administration of medical counter measures such as atropine sulfate, 2-PAM and diazepam or midazolam which are distributed in CHEMPACKS to personnel who may be exposed to OP toxicity (Bajgar, 2004; Reddy and Reddy, 2015). Benzodiazepines are the first line of treatment for seizures, but the evidence suggests that delayed administration does not prevent epileptogenesis. Midazolam, when compared with six other benzodiazepines, was found to be the most potent anticonvulsant (McDonough et al., 1999) which may explain the reduced mortality we saw in our model compared to diazepam. Several previous DFP studies with administration of benzodiazepines at least 40 minutes after exposure show early and persistent increases in gliosis, neurodegeneration and seizure activity (Kuruba et al., 2018; Wu et al., 2018; Putra et al., 2019; Gage et al., 2020; Rojas et al., 2020; Supasai et al., 2020). Other DFP studies considered the time of midazolam administration irrespective of the severity and duration of SE. One study administered midazolam at various time points following DFP and found a reduction in spiking activity, FJB, IBA1, and GFAP positive cells when midazolam was administered at 10-min, but the beneficial effects were not observed when it was administered after 40-min (Wu et al., 2018). Similarly, in our study, animals with > 31 min continuous CS during SE had widespread gliosis, reactive gliosis, and neurodegeneration in both hippocampal and extra hippocampal regions despite treating with midazolam. These groups of animals were administered midazolam at least 60-min post DFP. Notably, our Western blotting data did not always correlate with the IHC findings which suggests that the regional localization of the reactive-type glia may determine the pathological outcome. This highlights the importance of utilizing histochemical approaches to fully understand the extent of toxicity mediated by gliosis. As neuroinflammation and neurodegeneration are highly correlated with epileptogenesis, it is likely that some of these animals developed spontaneous recurrent seizures in < 7 days as observed during animal handling in several studies including our present study (Barker-Haliski et al., 2017; Rana and Musto, 2018; Andrew and Lein, 2021).

Interestingly, in contrast to animals with > 31 min of CS during SE, the rats with ∼20-min CS (in which midazolam was administered around 30-min after DFP), gliosis and neurodegeneration were localized primarily to the PC and AMY, but not the hippocampus. This is a **novel finding** demonstrating that SE duration and severity impacts the localization of injury. It is well established that the hippocampus, PC and AMY are all sensitive to injury by both DFP and other nerve agents (Aroniadou-Anderjaska et al., 2009; Figueiredo et al., 2018). This difference in localization might suggest that the insult is most severe or begins in the AMY/PC region. Researchers have identified a region in the piriform cortex called the “area tempestas” which is known to be a seizure trigger zone in response to chemoconvulsants such as bicuculline (a GABAergic antagonist) though it has not been studied in relation to OP toxicity (Ekstrand et al., 2001; Vaughan and Jackson, 2014). The quick onset of seizures following OP intoxication has made mapping seizure initiation difficult but there has been some progress in determining the most sensitive brain regions. Early work administered Soman or VX directly into various brain regions and found increased sensitivity primarily in the AMY, PC as well as dorsal hippocampus (McDonough et al., 1987; Tattersall, 2009). Similar studies with DFP may allow us to draw more relevant conclusions concerning regional sensitivity. Possibly there are differences in the concentration of muscarinic receptors or in the activity or regeneration of acetylcholinesterase which has been found to vary between brain regions (Bajgar et al., 2007; Prager et al., 2014). This could help to explain regional differences in gliosis and neurodegeneration. Interestingly, one study found that basal levels of acetylcholinesterase activity are higher in control amygdala compared to the hippocampus and piriform cortex which might suggest higher sensitivity to DFP exposure (Prager et al., 2013). This study also found lower levels of acetylcholinesterase activity in the basolateral amygdala of animals that developed SE after soman exposure compared to the animals that did not develop SE; this was not true of the piriform cortex or hippocampus. This finding suggests that reduction of acetylcholinesterase activity in the amygdala is critical for seizure initiation in this model.

The mechanisms of OP-induced SE leading to gliosis and neurodegeneration have been studied. Following the initiation of seizures due to inhibition of acetylcholinesterase, a rapid upregulation in glutamatergic activity contributes to the propagation of SE (McDonough and Shih, 1997; Weissman and Raveh, 2008; Kozhemyakin et al., 2010). Glutamate toxicity is a well-known cause of neuronal death (Lewerenz and Maher, 2015). Blocking a calcium plateau following OP toxicity, can prevent neurodegeneration (Deshpande et al., 2010). The excitotoxicity caused by increases in glutamate and calcium signaling leads to neuroinflammation through the activation of glial cells and the infiltration of peripheral macrophages, which also occurs in SE-induced injury due to compromised blood brain barrier integrity (Abbott and Friedman, 2012; de Furtado et al., 2012; Vezzani et al., 2013; Mendes et al., 2019). Importantly, the induction of SE, regardless of insult, leads to neurodegeneration and gliosis as demonstrated in this study and by others (Johnson et al., 2011; Walker and Sills, 2012; Reddy and Kuruba, 2013; Vezzani et al., 2013).

The regions analyzed in this manuscript are well studied and play specific roles in biological processes. The PC is a structure, consisting of several layers, primarily involved in olfactory processing and is also known to have some function in memory (Haberly, 1985; Linster and Hasselmo, 2001; Young et al., 2019). The amygdala is a structure consisting of several interconnected nuclei which are most classically known to play a role in emotion, particularly fear and anxiety but also cognition (Davis and Whalen, 2000; Rasia-Filho et al., 2000; Schaefer and Gray, 2007). Our analysis was focused primarily to the ventral piriform cortex and basolateral amygdala. Future work should more fully dissect the impact of DFP toxicity on specific regions in the AMY and PC as we did in the hippocampus which is primarily involved in memory and learning (Opitz, 2014; Fogwe et al., 2021). Due to the functional implications of insult to these regions, future work could use behavioral tests such as the Morris water maze, novel object recognition test and forced swim test to understand the impact of SE severity and duration on memory and anxiety. Also important, these regions as well as others beyond the scope of this study are highly interconnected. For example, anterograde tracers allowed researchers to detect excitatory projections from the basolateral amygdala to the ventral CA1 region of the hippocampus. These connections are well summarized by Vismer et al. (2015) as well as Yang and Wang (2017). This interconnectivity suggests that seizure induced injury in the ∼20-min group may spread from the PC and AMY region to other regions of the brain like the hippocampus over time (Yang et al., 2016).

In this study, 39% of the animals with ∼20 CS minutes did have SRS during handling which might suggest that injury to the AMY/PC region is sufficient to initiate epileptogenesis. In a rat pilocarpine model, the authors compared animals with 30-min convulsive SE to animals with 120-min convulsive SE (Bortel et al., 2010). There was an increase in both non-convulsive and convulsive seizures in animals with 120-min convulsive SE compared to the animals with 30-min convulsive SE which suggests that animals with lower duration of continuous CS during SE can still promote epileptogenesis but possibly not as severe as those with longer durations of CS during SE. In our study, there was no difference in the number of SRS between the groups with > 20-min CS but more EEG studies may reveal group differences. In 2008, a group developed a score to measure SE severity in humans called the “status epilepticus severity score” (STESS) which considered the level of consciousness, type of SE (partial, generalized), age and seizure history (Rossetti et al., 2008). Although it is not widely used clinics, there is a high correlation between STESS score and neurological outcome which implicates the importance of considering SE severity and duration in humans (Goyal et al., 2015).

In this study, to understand whether the DFP itself or DFP-induced SE severity impacts brain pathology, we investigated the animals that did not have continuous CS during SE (NCSE group) following exposure to DFP. These animals did show occasional convulsive seizures after DFP, but they did not persist like in the other groups. This group of animals might be comparable to another DFP study where “low responders” were compared with “high responders” wherein the average seizure score over two hours was used to categorize animals (González et al., 2020). The “low responders” did not show electrographic spikes but did show a transient calcium mineralization in the thalamus and neurodegeneration (labeled by FJC) in the somatosensory cortex 4 days post DFP. In another study, animals were exposed to small doses of DFP (400 μg/kg) for 5 days without the development of convulsive seizures (Phillips and Deshpande, 2016). These animals, however, showed neurodegeneration (FJC staining) in the dentate gyrus and an increase in anxiety when tested in the elevated plus maze. Interestingly, we did not find significant neurodegeneration (FJB positive cells) in the NCSE group compared to controls in CA1 and the piriform cortex. This might suggest that animals without a robust response to DFP are not subject to neuronal injury unlike in previous studies. Possibly, more sensitive measures may reveal more subtle neuronal damage in these animals though far less than those with continuous SE. These findings underscore the importance of considering the duration of CS in future analyses.

Status epilepticus severity and duration are important factors in determining the extent and localization of gliosis and neurodegeneration. Though we did not use EEG in this study for systematic seizure quantification, the quantification of behavioral CS duration during SE is a critical factor to test the efficacy of a disease modifier such as SAR. SAR is a Src family kinase inhibitor that has been tested in a variety of animal models and in clinical trials for neurological diseases and cancer (D8180C00004, NCT00704366, NCT04063124, NCT02167256, Kaufman et al., 2015). The mechanisms of Src family tyrosine kinase (SFK) inhibition in the mitigation of neurological disease are well studied. SFKs are a family of non-receptor binding proteins including Src, Fyn, Lck, Hck, Yes, Lyn, Blk, Fgr, and Yrk (Lieu and Kopetz, 2010). SFKs are implicated in a variety of neurological processes including cell proliferation, cell differentiation, cell migration, apoptosis, metabolism, and long term potentiation (Lu et al., 1999; Purcell and Carew, 2003; Parsons and Parsons, 2004; Nie et al., 2020). Following neurological insult, SFKs, more specifically Fyn and Src, are upregulated and can initiate signaling cascades that contribute to neuroinflammation and hyper-excitability (Kaufman et al., 2015; Liu et al., 2016; Nygaard, 2018; Panicker et al., 2019). In neurons, Fyn can modulate both NMDARs, metabotropic glutamatergic receptors, and gamma aminobutyric acid receptors (GABAR) (Salter and Kalia, 2004; Knox and Jiang, 2015; Wang et al., 2016; Jin et al., 2017; Putra et al., 2020b). This regulation can lead to excitotoxicity and to the development of epilepsy. Another study showed that Src is cleaved by calpains (Hossain et al., 2013). Calpains can be upregulated following neurological injury including seizures which would implicate that there is an increased prevalence of truncated Src following OP induced SE (Vosler et al., 2008; Lam and González, 2019). The truncated form of Src was sufficient to induce neuronal death in primary cortical neurons (Hossain et al., 2013). In glial cells, phosphorylated Fyn, an SFK highly upregulated in the brain, leads to the phosphorylation of PKCδ and NFκB mediated transcription of proinflammatory cytokines and inducers of oxidative stress (Panicker et al., 2015, 2019). Inhibition of SFKs can attenuate microglial activation (Manocha et al., 2015; Socodato et al., 2015). Another study in a mouse microglia cell line demonstrated the complement receptor 3 mediated activation of Src leading to the phosphorylation of NADPH oxidase 2 (NOX2) subunits, contributing to oxidative stress (Hou et al., 2018). Furthermore, Src is also implicated in the phosphorylation of inducible nitric oxide synthase (iNOS) in cancer cells though it has not yet been explored in the context of neurological disease (Tyryshkin et al., 2010). Fyn can also phosphorylate p130Cas leading to translocation of the Pyk2/paxillin complex to the membrane (Fan et al., 2017). This complex is essential for microglia migration which suggests the role of SFKs play in both microglia activation and chemotaxis.

Our lab and others have demonstrated the specific role of Fyn kinase in epileptogenesis. Early work showed that mice with a null mutation in *fyn* had a lower rate of amygdala kindling (Cain et al., 1995) while mice overexpressing *fyn* showed a reduced seizure threshold (Kojima et al., 1998). Our work showed that *fyn* knockout mice had reduced SE severity following repeated low doses of KA although there was no difference in the amount of KA needed to initiate the first CS (Sharma et al., 2018). In the mouse pentylenetetrazole model, our lab showed that *fyn* knockout mice had a reduction in SE severity compared to wild type mice though the difference was not significant (Putra et al., 2020b). Importantly, the *fyn* knockout mice treated with pentylenetetrazole did not have significant neurodegeneration compared to *fyn* knockout mice without pentylenetetrazole which suggests mitigation of Fyn is neuroprotective. Although knockout studies are useful, other studies with pharmacological inhibition of SFKs allow us to fully understand the epileptogenic potential. One study used PP2, a SFK inhibitor *in vitro* and found a reduction in epileptiform discharges in hippocampal CA3 slices stimulated with a potassium blocker (Sanna et al., 2000). In a mouse KA model, SAR pre-treatment (25 mg/kg, single dose), 4 h before KA challenge, significantly reduced the initial SE severity (Sharma et al., 2018). In the rat KA model, SAR post-treatment led to significantly less SRS, and significant reduction in gliosis and neurodegeneration (Sharma et al., 2018, 2021). Another study using the mouse pilocarpine model, showed that SAR reduced the spiking frequency, spontaneous seizures, and neuronal loss (Luo et al., 2021). Given these studies, we decided to test SAR in the rat DFP model.

We chose SAR over the other Src family kinase inhibitors as SAR inhibits the function of the ATP binding cassette transporter, ABCG2 which allows it to cross the blood brain barrier effectively (Kast and Focosi, 2010; Tyryshkin et al., 2014; Mittapalli et al., 2016). SAR’s safety, tolerability, and pharmacokinetics in phase I clinical trials confirmed that the drug at 125 mg was safe and well tolerated in patients with advanced solid tumors (Fujisaka et al., 2013). In another cancer study, SAR was used chronically at 175 mg (Baselga et al., 2010). SAR was also tested in healthy human volunteers in single doses up to 1000 mg and multiple doses up to 250 mg QD for 14 days (Eastell et al., 2005). The rationale for choosing the 25 mg/kg dosing regimen was based on the findings from our two previous studies (Sharma et al., 2018, 2021). Notably, the human equivalent dose is around 4.05 mg/kg or around 240mg for an average weight human which is slightly above most doses used in the clinical trials mentioned above (Nair and Jacob, 2016). Since this dosing regimen was well tolerated in the KA and pilocarpine models, we tested a similar dosing regimen, in the DFP model with some modification.

We first tested SAR in animals with > 60-min CS and found significant weight loss and mortality in animals (4 out of 9) treated with SAR compared to vehicle treated animals. Due to the early mortality and weight loss, we decided to change the dosing regimen for the last four days from 25 mg/kg to 20 mg/kg. This mortality and morbidity could be due to the combined effects of SE severity, DFP-induced gut changes, and the high dose of SAR. The twice a day dosing is well above the human equivalent dose that is normally administered in clinical trials. We hypothesize that lower and less frequent doses may increase efficacy and lower toxicity. We did not see this degree of mortality in the animals with ∼20-min CS which suggests there is an interaction between SE severity, duration, and SAR toxicity. Some clinical trials with SAR in advanced/terminals stages of the disease reported adverse effects. For example, one study in patients with metastatic breast cancer administered 175 mg SAR daily for 28 days (Gucal et al., 2011). 33% of patients in this study experienced at least one adverse effect during the treatment period such as fatigue, nausea, and hyponatremia. Another study in patients with probable Alzheimer’s dementia administered SAR in ascending doses from 50 mg to 125 mg and reported the most common adverse events to be diarrhea, headache, fatigue and nausea (Nygaard et al., 2015). In an *in vitro* rat liver microsomes study, a LC-MS/MS method detected potential reactive metabolites of SAR such as cyanide adducts and GSH (an anti-oxidant enzyme) conjugate (Attwa et al., 2018). These reactive metabolites might covalently bind to proteins or other macromolecules in the body to cause toxicity and adverse effects. In another study, intraperitoneally administered SAR in rats found oxidative metabolites in urine and bile (Chen et al., 2016). However, it is unclear whether these are toxic metabolites or normal biodegradation products of SAR. More research is needed to fully understand the mechanisms of SAR toxicity in the DFP model.

Saracatinib is bio-activated by P450 3A4, a predominant liver and intestinal enzyme responsible for the metabolism of many of pharmacological agents (Ioannides and Lewis, 2004; Chen et al., 2016). Notably, changes in the regulation of drug metabolizing enzymes such as P450 34A can lead to hepatotoxicity and gastrointestinal toxicity (Tao et al., 2020). We did not measure the enzyme levels or test hepatotoxicity and gastrointestinal toxicity, which are beyond the scope of this study. However, it is speculative that the toxicity due to higher dosing regimen in this study could be due to these factors, which requires further investigation. Interestingly, SAR metabolites also inhibit its own metabolizing enzyme, maybe to serve as a self-limiting process to limit SAR toxicity (Chen et al., 2016). However, P450 3A4 is also required for metabolism of several drugs used in clinics including benzodiazepines such as midazolam, which was used in this study to control seizures (Li et al., 2021). In this study, SAR was administered two hours after treating animals with midazolam. Considering midazolam’s half-life (body clearance) is < 2 h (Hovinga et al., 1992), it is unlikely that the toxicity in this study was due to SAR-midazolam interaction. It may also be important to consider the other medical countermeasures used in the model. ATS has a plasma half-life of around 40-minutes when administered intraperitoneal (Harrison et al., 1974) while 2-PAM was found to have a tissue clearance of approximately 10.9 ml/min (Green et al., 1986). The pharmacokinetics of the drug may change when administered intramuscularly or after exposure to an organophosphate. For example, one study found that atropine metabolism was inhibited in people who had ingested organophosphate pesticides (Van der Meer et al., 1983). Furthermore, we only administered midazolam, ATS and 2-PAM once while SAR was administered several times so it is unlikely that these MCMs influenced the metabolism of SAR at later doses.

Considering the high mortality in > 60-min continuous CS group, we thought that the severity of seizures had also contributed to the SAR-induced toxicity/morbidity. We, therefore, focused the rest of our analysis on the brain IHC in the group with ∼20-min CS. In this group, we maintained the same dosing regimen and increased the dose by 5 mg/kg for the last four days of the treatment since the SE severity was almost 3 times less severe than > 60-min CS group. In studies such as this, the initial SE severity between the vehicle and the test drug must be balanced to investigate the real therapeutic effects of the intended test drug. There was no difference in the number of minutes the VEH treated animals and SAR treated animals spent in CS during SE in this study.

Saracatinib treatment in this study started two hours after midazolam administration, and reduced gliosis and neurodegeneration in the PC and AMY. The reduction in neurodegeneration was significant and the reduction was trending for gliosis likely because the treatment duration was short unlike longer duration in other SAR studies (Luo et al., 2021), or a longer washout maybe required to observe the desired mitigating effects. It is also likely that there could be a more robust reduction in gliosis if animals were sacrificed before SAR is metabolized. Previous studies have shown that the half-life of SAR in humans is 40 h while in mice it is about 16 h (Kaufman et al., 2015). It is likely that the half-life of SAR is different in rats exposed to OPs, which requires further investigation. Of note, none of the SAR treated animals in this study had SRS post-DFP during animal handling in contrast to the vehicle group which had several SRS in the first week of DFP exposure. This is in alignment with previous work that showed a reduction in SRS using EEG (Sharma et al., 2018, 2021). We also observed CS during the first 24h post-DFP (not shown in the heat maps) in both the VEH and the SAR groups which could be due to the residual effects of DFP in the brain, but SRS were not observed in SAR group thereafter. Other SE severities and durations should be tested with alternative dosing regimens to fully evaluate the disease-modifying potential of SAR.

The mitigation of neurodegeneration and SRS and a reduction in gliosis by SAR is consistent with previous studies in other models from our laboratory and others (Sharma et al., 2018, 2021; Luo et al., 2021). In future studies, SAR should be tested in OP models to determine the effect on spiking activity and electrographic seizures as we did in others models. SAR has also been studied in clinical trials for patients with Alzheimer’s dementia (Nygaard et al., 2015; Van Dyck et al., 2019). These studies had limited outcomes which might suggest that SAR’s efficacy is dependent on administration at an early stage of the disease onset. However, in a mouse model of AD, long-term SAR treatment for about 4 weeks, had significant disease-modifying effects implying that the duration of treatment is also a critical factor to determining the efficacy of SAR (Smith et al., 2018). It is also important to address the limitations in using animal models in comparison to humans as preclinical trials are not always predictive of toxicity or efficacy in clinical trials (Bracken, 2009; Van Norman, 2019). It is also possible that SAR might have greater efficacy when administered with other anti-epileptic drugs. Overall, these observations from our study suggest that SAR might be useful in mitigating epileptogenesis but a long-term treatment is required to determine its efficacy in the DFP model.

## Conclusion

In summary, we demonstrated the impact of SE severity and duration on gliosis and neurodegeneration. We found that animals with > 31 min continuous CS during SE had widespread neural injury in both hippocampal and extra-hippocampal regions while animals with only ∼20-min continuous CS during SE had neural injury localized primarily to the PC and AMY. Animals with no continuous CS during SE did not show significant changes in gliosis and neurodegeneration. Future work could focus on other regions on the brain in addition to the ones we focused on in this study. We also tested the disease modifier, SAR, for tolerability in animals with > 60-min and ∼20-min continuous CS. We found a reduction of gliosis but significant mitigation of neurodegeneration and SRS, which suggests SAR may be useful as a mitigation strategy against OP toxicity. This study suggests that a SAR dosing regimen to achieve significant disease-modifying effects is dependent on the severity of SE. Future studies should focus on dose optimization based on initial severity and duration of CS during SE, induced by DFP or its OP analogs such as soman. In addition to dose optimization, future studies should also determine the optimal timing of SAR administration following DFP intoxication. In this study, we administered SAR 2 h following midazolam; earlier administration may be more efficacious. In this study we focused primarily on the short-term impact of DFP and SAR (24 h after the last dose of SAR). Future studies could determine the long-term impact of SAR on DFP induced toxicity as well as focus on other epileptogenic parameters such as seizures (assessed through EEG) as well as other behavioral changes in learning, memory, and anxiety. In conclusion, in this study, we determined the impact of SE severity following DFP intoxication and the disease-modifying potential of SAR in mitigating gliosis and neurodegeneration.

## Data Availability Statement

The raw data supporting the conclusions of this article will be made available by the authors, without undue reservation.

## Ethics Statement

The animal study was reviewed and approved by Iowa State Animal Care and Use Committee.

## Author Contributions

MGag conducted animal experiments, collected and analyzed data, and drafted and edited the manuscript. MP conducted animal experiments and collected data. CG-E, MGo, LW, and MGar participated in cell counting and sectioning for immunohistochemistry experiments. TT conceptualized the idea, secured the funding for the project, designed the study, and edited the manuscript. All authors edited the manuscript.

## Conflict of Interest

The authors declare that the research was conducted in the absence of any commercial or financial relationships that could be construed as a potential conflict of interest.

## Publisher’s Note

All claims expressed in this article are solely those of the authors and do not necessarily represent those of their affiliated organizations, or those of the publisher, the editors and the reviewers. Any product that may be evaluated in this article, or claim that may be made by its manufacturer, is not guaranteed or endorsed by the publisher.
