# Supplementary material for: Differential Impact of Severity and Duration of Status Epilepticus, Medical Countermeasures, and a Disease-Modifier, Saracatinib, on Brain Regions in the Rat Diisopropylfluorophosphate Model
Source: Front Cell Neurosci. 2021 Oct 15;15:772868. doi: 10.3389/fncel.2021.772868 (PMC8555467; doi:10.3389/fncel.2021.772868)

Supplementary Material

## Supplementary Figures (images 1 and 2) Supplementary Figure 1. Full blots for IBA1 and GFAP from the hippocampus and piriform cortex/ amygdala. Green filter indicates IBA1 and GFAP while the red filter indicates B-actin used for normalization.
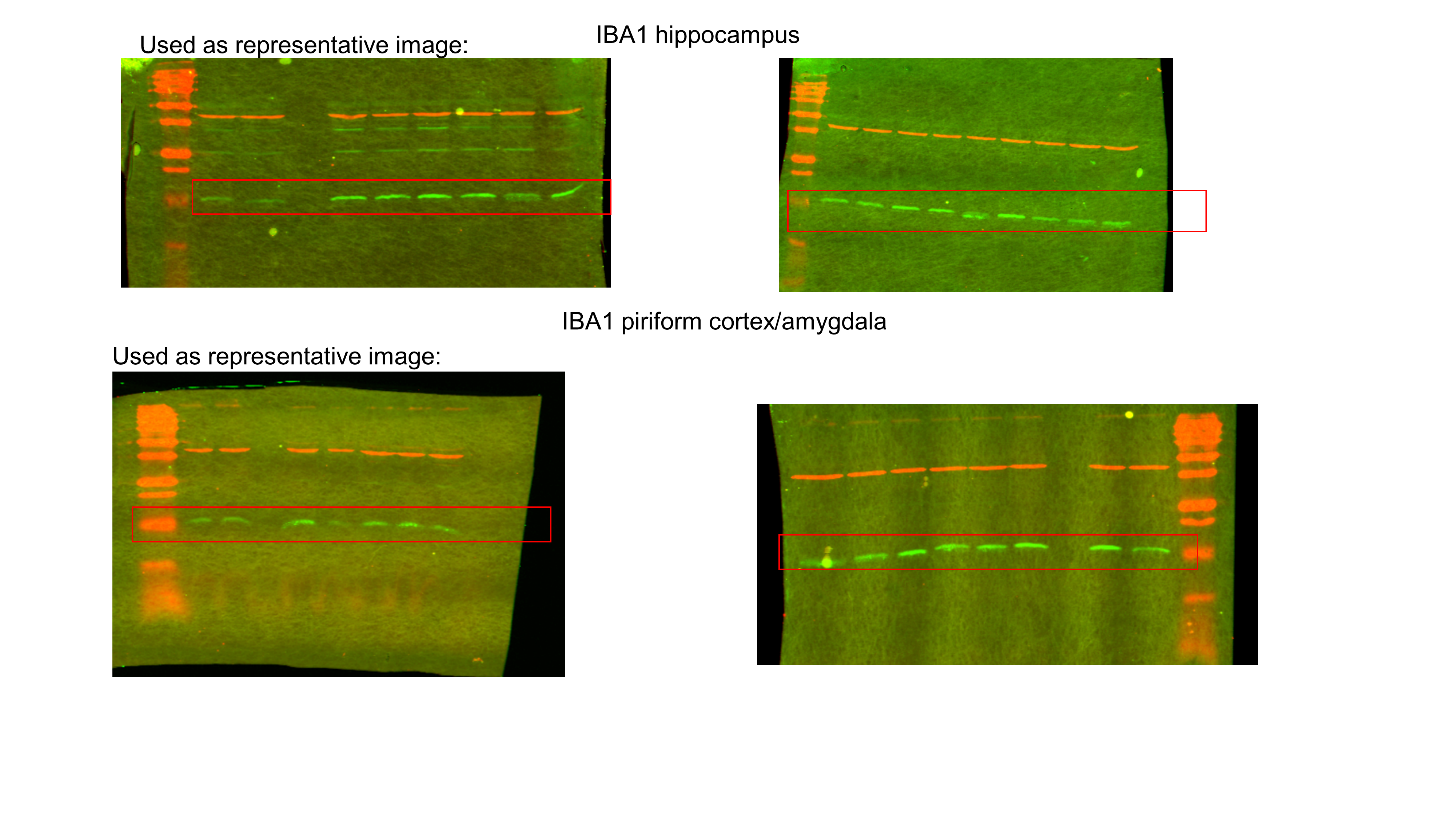


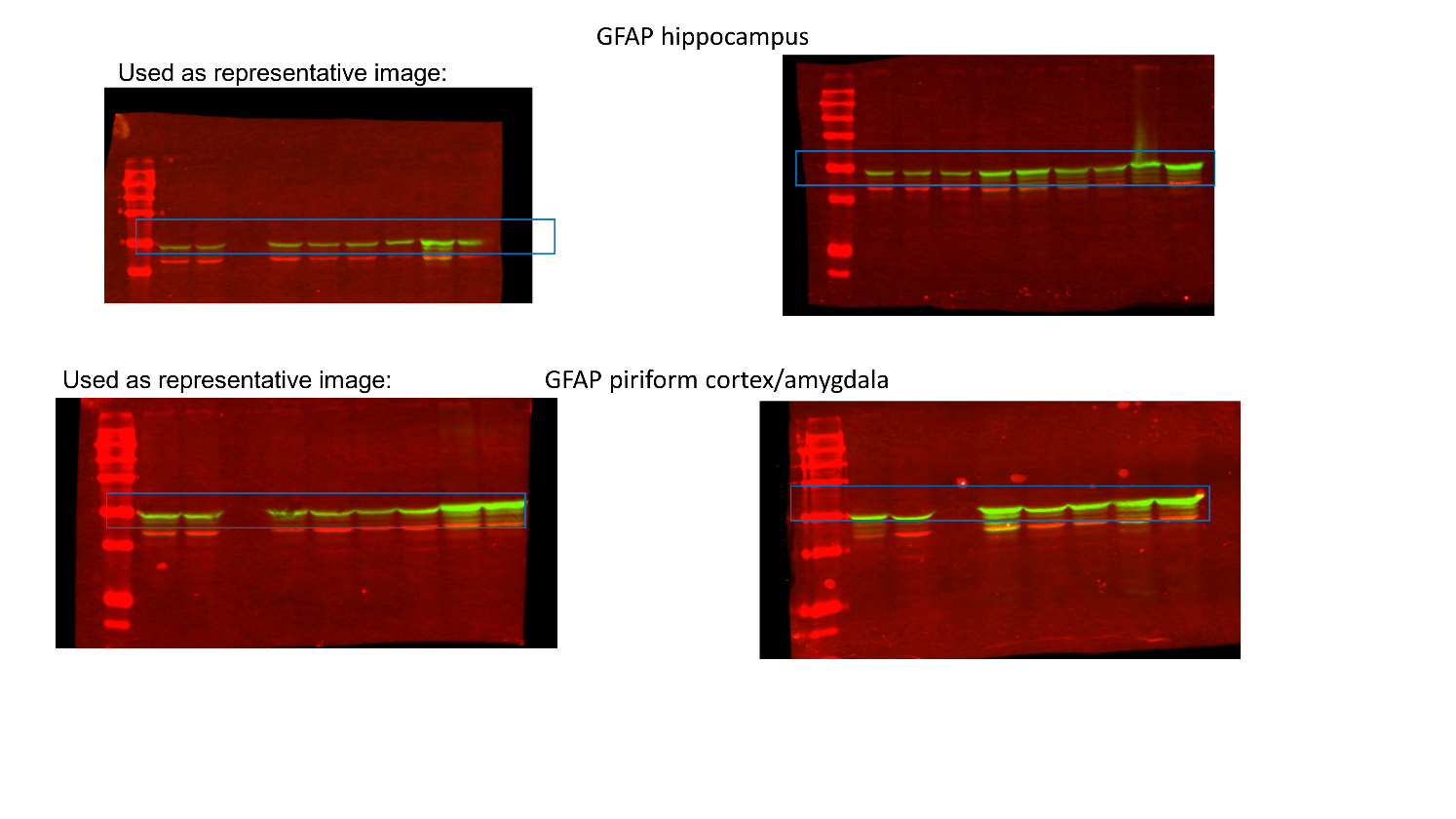

Supplement: Supplementary file 1 [file Data_Sheet_1.docx]
